# Cryo-electron microscopy of adipose tissue extracellular vesicles in obesity and type 2 diabetes mellitus

**DOI:** 10.1371/journal.pone.0279652

**Published:** 2023-02-24

**Authors:** Valentina V. Miroshnikova, Kseniya V. Dracheva, Roman A. Kamyshinsky, Evgeny V. Yastremsky, Luiza A. Garaeva, Irina A. Pobozheva, Sergey B. Landa, Kristina A. Anisimova, Stanislav G. Balandov, Zarina M. Hamid, Dmitriy I. Vasilevsky, Sofya N. Pchelina, Andrey L. Konevega, Tatiana A. Shtam

**Affiliations:** 1 Petersburg Nuclear Physics Institute named by B.P. Konstantinov of National Research Centre “Kurchatov Institute”, Gatchina, Russian Federation; 2 Pavlov First Saint Petersburg State Medical University, St. Petersburg, Russian Federation; 3 National Research Centre “Kurchatov Institute”, Moscow, Russia; Nippon Medical School, JAPAN

## Abstract

Extracellular vesicles (EVs) are cell-derived membrane vesicles which play an important role in cell-to-cell communication and physiology. EVs deliver biological information from producing to recipient cells by transport of different cargo such as proteins, mRNAs, microRNAs, non-coding RNAs and lipids. Adipose tissue EVs could regulate metabolic and inflammatory interactions inside adipose tissue depots as well as distal tissues. Thus, adipose tissue EVs are assumed to be implicated in obesity-associated pathologies, notably in insulin resistance and type 2 diabetes mellitus (T2DM). In this study we for the first time characterize EVs secreted by visceral (VAT) and subcutaneous adipose tissue (SAT) of patients with obesity and T2DM with standard methods as well as analyze their morphology with cryo-electron microscopy. Cryo-electron microscopy allowed us to visualize heterogeneous population of EVs of various size and morphology including single EVs and EVs with internal membrane structures in samples from obese patients as well from the control group. Single vesicles prevailed (up to 85% for SAT, up to 75% for VAT) and higher proportion of EVs with internal membrane structures compared to SAT was typical for VAT. Decreased size of single and double SAT EVs compared to VAT EVs, large proportion of multilayered EVs and all EVs with internal membrane structures secreted by VAT distinguished obese patients with/without T2DM from the control group. These findings could support the idea of modified biogenesis of EVs during obesity and T2DM.

## Introduction

Obesity is a major risk factor for the development of metabolic syndrome, hypertension, cardiovascular disease, dyslipidemia, insulin resistance, and diabetes mellitus [1]. The role of adipose tissue (AT) released extracellular vesicles (EVs) in obesity-associated pathologies, notably in insulin resistance, has been actively discussed in recent years [2,3].

EVs are produced by cells of all known organisms and are important for cell-to-cell communication and physiology. EVs, which are vesicles surrounded by a phospholipid bilayer, form heterogeneous population classified as exosomes, microvesicles (MVs), and apoptotic bodies based on their size and mechanism of formation [1,4]. Exosomes are considered to be of endocytic origin and released upon fusion of multivesicular bodies (MVB) and the plasma membrane, MVs are directly derived from the plasma membrane and apoptotic bodies are formed exclusively during programmed cell death [4]. EVs can transmit a broad range of molecular signals through transfer of different cargo such as lipids, proteins and nucleic acids, including microRNAs, to recipient cells and the extracellular environment [3]. Such ubiquity establishes EV release as a fundamental process required for cellular communication in both normal and pathological conditions [4]. Thus, EVs have received much interest for clinical application as diagnostic biomarkers and therapeutic carriers of bioactive molecules [5].

Secretion as well as composition of EVs might be altered in obesity and diabetes mellitus [1]. Obesity is associated with an increase in the level of plasma EVs [6–8]. Adipose tissue EVs could regulate metabolic and inflammatory interactions inside adipose tissue depots as well as distal tissues [9,10]. For example, adipocytes were found to secrete lipid-laden EVs expressing the lipid droplet-associated protein perilipin 1, phospholipids, neutral lipids, and free cholesterol that are taken up by AT macrophages and can promote macrophage polarization into M1 phenotype [11]. In diabetes mellitus, EVs may stimulate endothelial cells to transform from normal phenotype into a diabetic phenotype [1]. It could be expected that AT EVs are likely involved in obesity-associated comorbidities. Still there is only limited information on the morphology and composition of EVs secreted directly by human AT [12,13].

Great morphological diversity has been described regarding EVs found in body fluids such as blood plasma, breast milk, ejaculate, cerebrospinal fluid [14–19]. All these studies established cryo-EM as the most suitable technique to study the morphology of EVs because it provides high spatial resolution and the ability to image EVs preserved in a near-native state using vitrification, without any staining or chemical fixation procedures [20–23]. Still today cryo-EM structure of AT EVs was not studied. Analysis of morphology of AT EVs in obesity and type 2 diabetes mellitus (T2DM) by cryo-EM could widen our understanding of the pathophysiological role of AT EVs in these diseases. Here we characterize EVs secreted by visceral and subcutaneous AT of patients with obesity and T2DM with standard methods as well as analyze their morphology with cryo-EM.

## Materials and methods

### Study participants

Patient groups were filled with obese subjects with or without T2DM who underwent a bariatric surgery and had body mass index (BMI)>35. Type 2 diabetes mellitus diagnosis was based on clinical and laboratory characteristics as per the 1999 WHO criteria for diabetes classification and diagnosis [24]. Patients with the following characteristics were included: fasting plasma glucose levels ≥7.0 mmol/L or 2 h post-challenge glucose levels in an oral glucose tolerance test ≥11.1 mmol/L. Control group was formed by normoglycemic subjects without obesity and T2DM who was selected from a convenience sample of patients undergoing unrelated abdominal procedures. Patient data is represented in Table 1.

**Table 1:** Baseline demographic, clinical and biochemical data of patients.

Studied groups	Obesity with type 2 diabetes mellitus N = 7	Obesity without type 2 diabetes mellitus N = 6	Control group without obesity or type 2 diabetes mellitus N = 9
Age	47±9	39±11	41±8
Male/Female	4/3	1/5	3/6
Body mass index	47.5±7,2*	40.9±5,5*	24.0±3,0
Weight	108–173	100–145	60–95
Glucose, mmol/L	7.3 (5.7–9,6)¥§	5.6 (5.4–5.9)¥	5.0 (4.3–5.5)
Cholesterol, mmol/L	5.5±0.6	4.7±0.9	nd
HDL, mmol/L	1.3±0.1	1.3±0.2	nd
LDL, mmol/L	3.0±0.8	2.5±0.8	nd
Triglycerides, mmol/L	2.6±1.1	2.1±1.2	nd

Notes: nd–not determined

* p = 0.000 vs control group

¥ p<0.05 vs control group

§ p<0.05 vs obese without type 2 diabetes mellitus.

The study protocol is in accordance with the Declaration of Helsinki and was approved by the local ethics committee of Pavlov First Saint-Petersburg State Medical University, Saint-Petersburg, Russian Federation (protocol 259 by 2022 February 28). Written informed consent was given by each participant.

### Adipose tissue cultivation and extraction of extracellular vesicles

Visceral adipose tissue (VAT) and subcutaneous adipose tissue (SAT) were excised during surgery from the omentum and the anterior abdominal wall incision site, respectively, immediately placed into Hank’s solution and transported to the laboratory.

VAT and SAT samples (1–2 g) were washed with phosphate buffered saline (PBS), cut into 1–4 mm pieces, transferred to petri dish containing DMEM/F12 medium with 10% EV-free serum (Fetal Bovine Serum, exosome-depleted, Thermo Fisher Scientific, A2720803) supplemented with 1% gentamicin and incubated for 12 h. The culture supernatant was prepared via serial centrifugations and filtration, specifically it was centrifuged at +4°C at 300g for 10 minutes, 3,500 g for 30 min, 10,000 g for 30 min; afrerwards it was filtered through 0,22 syringe PES filter to remove lipids, cells and cellular debris before ultracentrifugation. Culture medium prepared in this way was frozen in liquid nitrogen and stored at -80°C. 100 ml of pooled culture medium (thawed on ice) was subjected to ultracentrifugation at 110,000 g and +4°C to pellet EVs (Optima L-90K centrifuge, Ti45 rotor (Beckman Coulter)). EVs were washed by PBS and centrifuged again at 110,000 g and +4°C (Optima L-90K centrifuge, SW 55Ti rotor, (Beckman Coulter)) before the final EVs pellet was resuspended in 100 μL of PBS, and aliquots were frozen in liquid nitrogen and stored with subsequent storage at −80°C for further analysis.

### Nanoparticle Tracking Analysis (NTA)

The size and concentration of EVs were determined by NTA using the NTA NanoSight LM10 analyzer, equipped with a 405 nm laser (Nano-Sight, Malvern Instruments) and a C11440-5B camera (Hamamatsu Photonics K.K.). Before NTA measuring, an aliquot of the isolated EVs was thawed at room temperature and diluted with deionized water 1,000, 10,000, 100,000 times and injected in the sample chamber with sterile syringe. Recording and data analysis were performed using the NTA software 2.3. Particles were captured by recording 30s video at room temperature and following parameters: camera level—16, low threshold—0, high threshold—2015. Received videos (750 frames captured with 10–40 particles/per frame) were analyzed at detection threshold value of 8 and minimal expected size. The measurements were carried out three times. Software used identifies and tracks individual nanoparticles moving under Brownian motion and relates the movement to a particle size according to the Stokes-Einstein formula. So, the average hydrodynamic diameter, the mode of distribution, the standard deviation, and the concentration of vesicles in the suspension were determined.

### Dynamic Light Scattering (DLS)

Unprocessed EV preparations as well as after preliminary depletion of unnecessary particles by immunoprecipitation with anti-CD63 antibodies were subjected for DLS as described earlier [25,26]. This approach allows to distinguish particles by size (using hydrodynamic radius). The measurements were carried out using a laser correlation spectrometer DLS (INTOX MED LLC, St. Petersburg, Russia) with a heterogeneous measurement scheme. Mathematical processing of the obtained data was carried out using the algorithm [27] using the QELSspec (version 3.4) software package, Gatchina, Russia.

### Western blotting

EVs were lysed 1:1 in ice-cold RIPA buffer containing 50 mM Tris-HCl (pH 8.0), 150 mM NaCl, 1% Triton X-100, 0.5% sodium deoxycholate, 0.1% SDS and protease inhibitor cocktail (Roche). The lysate was centrifuged at 14,000 g for 15 min at 4°C, then supernatant was carefully aspirated into a new tube. Protein concentrations were determined using the Micro BCA protein assay (Pierce). A mass of 5 μg (EVs) and 10 μg (AT lysate as a control) protein per lane was separated using 8% SDS-PAGE gels. Proteins were transferred to PVDF membranes (Millipore) and pre-incubated with 5% skim milk in PBS. The blots were incubated with rabbit polyclonal anti-СD63 (1:1000; ab216130, Abcam) and anti-FABP4 (1:1,000; PA5-30591, Thermo Fisher Scientific) primary antibodies diluted in 1% skim milk in PBST (0.05% Tween 20 PBS) to prevent non-specific binding and followed by anti-rabbit HRP-conjugated secondary antibodies (1:3,000; ab6721, Abcam). Proteins were visualized using an ECL Western Blotting Detection Reagent (Amersham) using ChemiDoc Imaging system (BioRad).

### Cryo-EM

Cryo-EM was used for direct visualization of vesicles and their morphological examination. To prepare samples for cryo-EM study lacey carbon EM grids were glow-discharged (30 s, 25 mA) in Pelco EasiGlow system. 3 μL of the sample aqueous solution were applied onto the carbon side of EM grid, which was then blotted for 2.0 s using filter paper and plunged into the precooled liquid ethane with Vitrobot Mark IV (FEI, USA). This procedure results in embedding the samples in a thin layer of electron transparent amorphous ice preserving them in native state and protecting from electron beam damages. Plunge-frozen samples were imaged in a cryogenic transmission electron microscope Titan Krios 60–300 (FEI, USA), equipped with highly sensitive direct electron detector (DED) Falcon II (FEI, USA) and Cs image corrector (CEOS, Germany) at accelerating voltage of 300 kV. Acquisition of cryo-EM images was performed using EPU software (FEI, USA) in low-dose mode to minimize radiation damage, most of the images collected for analysis was taken with 7.45 Å per pixel, small number of images were taken at double the magnification. EVs feature measurement was performed in Fiji [28].

### Statistical analysis

Conformity of findings to normal distribution was tested using the Shapiro-Wilk test. To assess differences between groups, the Mann–Whitney test was used. Differences in the proportion of EV types (multilayered, total with internal membrane structures) were assessed by contingency tables accomplished using Fisher’s exact test. The level of significance was set at p < 0.05. Statistical analysis was performed using SPSS 17.0 software. Clinical and experimental data are presented as the mean ± the standard deviation (SD) or the median (min-max) depending on the distribution.

## Results

### Characterization of adipose tissue EVs by NTA, western blotting and DLS

**Fig 1:**
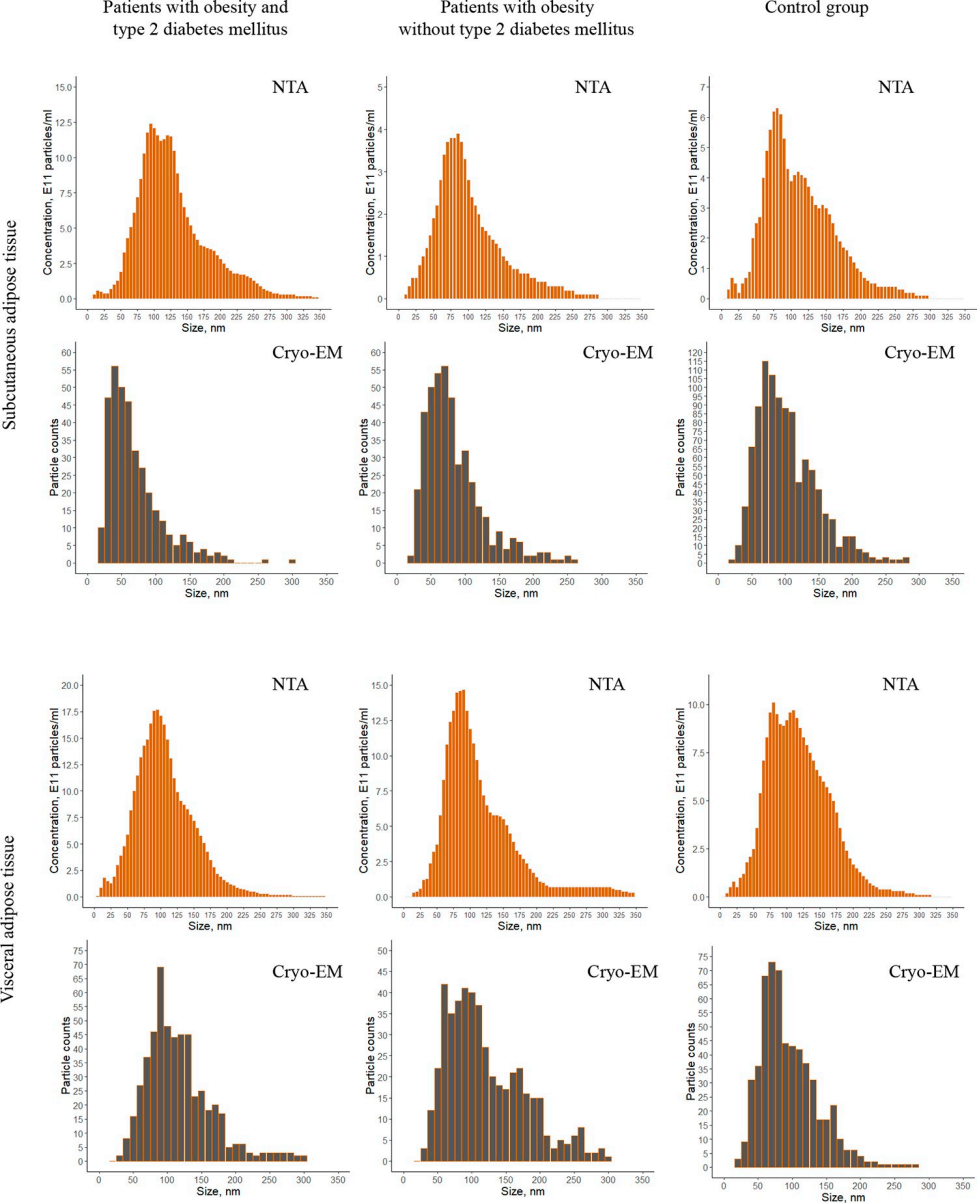
Size distribution of subcutaneous and visceral adipose tissue extracellular vesicles assessed by NTA and cryo-EM.

NTA was conducted for AT EVs of obese patients with/without T2DM and individuals without obesity and T2DM. On the whole EVs yielded concentration from 6.8*10^12 to 2.9*10^13 particles/mL with an average modal size of 89±17 nm for SAT EVs and 84±10 nm for VAT EVs. Parameter D90 (the diameter at which 90% of the samples’ mass is comprised of particles with a diameter less than this value) was estimated as 186±17 nm and 176±16 nm for SAT and VAT EVs, respectively. The characteristic sizes of all EV preparations are shown in the Table 2. NTA size distribution diagrams for all groups studied are presented in Fig 1. Purity of EV preparations was assessed as the ratio of NTA measured particle number per μg of protein which introduces contaminating protein [29]. These data are represented in the Table 2 and demonstrate receivable purity level for ultracentrifugation.

**Table 2:** Characteristic of EV preparations for cryo-EM.

Sample number	Sample type	D90, nm	Concentration of EVs, particles/mL	Purity, particles per μg of protein
1 2	Obesity without T2DM SAT VAT	182±19 176±18	6.8x10^12 2.6x10^13	1.1x10^10 8.9x10^9
3 4	Obesity with T2DM SAT VAT	192±15 172±16	2.3x10^13 2.9x10^13	4.1x10^9 5.2x10^9
5 6	Control group SAT VAT	176±11 184±19	1.2x10^13 2.3x10^13	1.0x10^10 1.7x10^10

DLS analysis was proceeded for unprocessed EV samples and after CD63-positive particles depletion (S1 Fig). DLS analysis of unprocessed samples resulted in two distinct peaks corresponding to vesicle hydrodynamic radius in the range of 20–30 nm (red peak, contribution to scattering of 30–40%) and 120 nm (blue peak, contribution to scattering of 50–65%), respectively, without differences of size distribution between different EV preparations. DLS analysis of CD63+ particles depleted samples demonstrated contamination of samples by small CD63- particles about 1% (S1 Fig).

**Fig 2:**
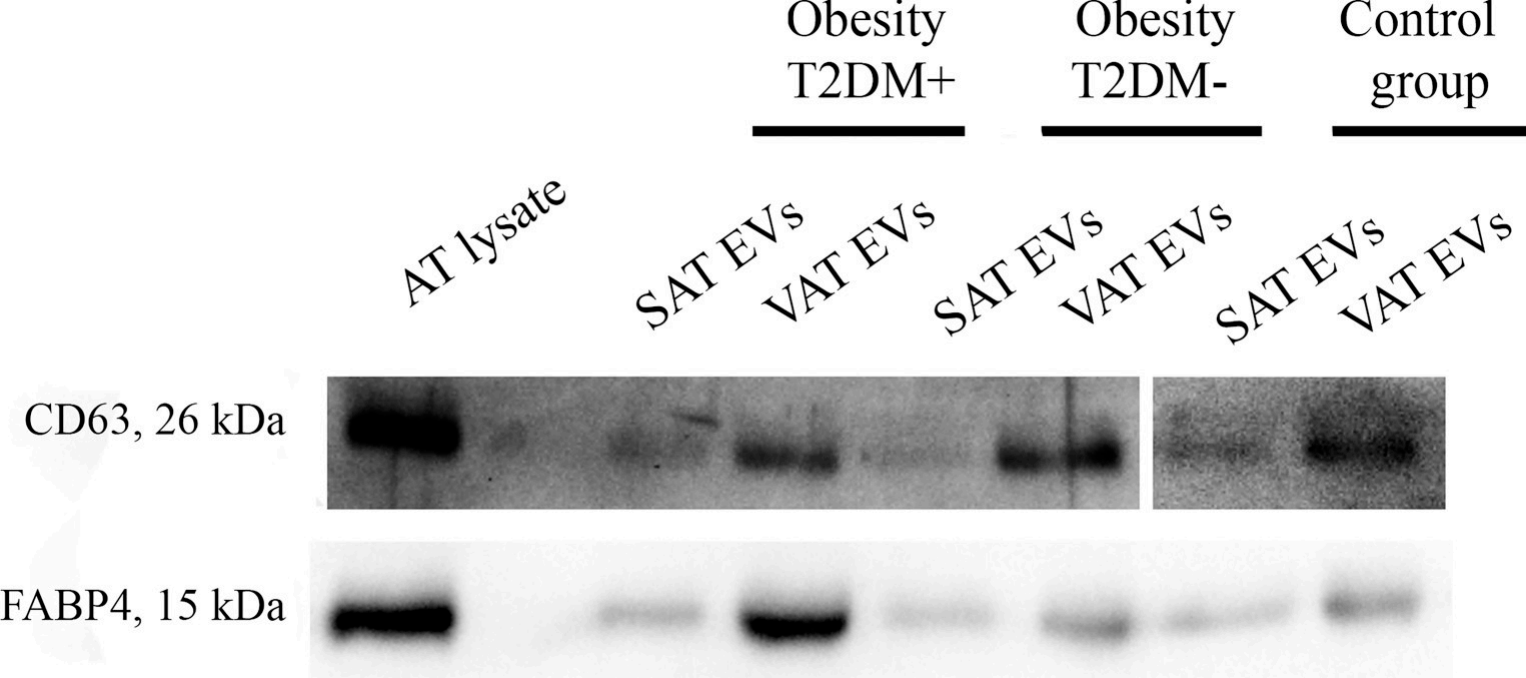
Western blot analysis of the CD63 as common exosomal marker and FABP4 as adipose tissue specific marker. The origin bands are presented in supplementary files. Abbreviations in the figure: AT–adipose tissue, SAT–subcutaneous AT, VAT–visceral AT, EVs–extracellular vesicles, T2DM–type 2 diabetes mellitus.

All EV preparations were analyzed by Western-blot. The isolated particles contained canonical exosomal marker CD63 as well as adipocyte-specific fatty acid binding protein 4 (FABP4) (Fig 2). Unprocessed western blot images can be found in S1 File.

### Morphological characterization of adipose tissue EVs by сryo-EM

Sufficient number of EVs were counted and analyzed in series of microphotographs: from 334 to 1014 EVs depending on the study group. EVs of various sizes and morphology including EVs with internal membrane structures were observed by cryo-EM. More than 90% of EVs were identified as exosome-like vesicles due to the clear presence of a lipid bilayer. As expected, most of the vesicles were round, but elongated vesicles were also detected.

**Fig 3:**
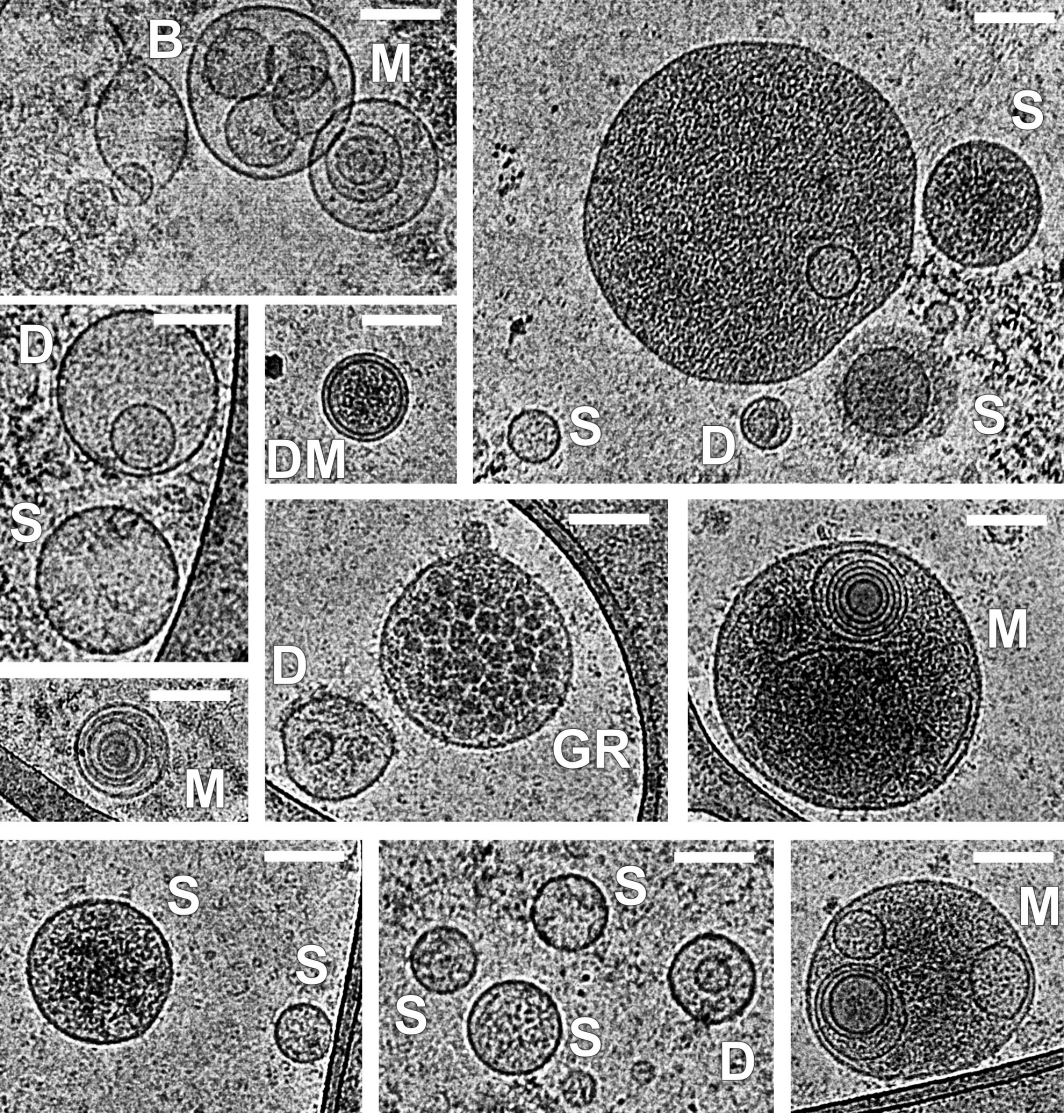
Cryo-EM images of EVs isolated from SAT and VAT culture mediums of obese patients with and without T2DM. Various morphological types of extracellular vesicles have been identified: Single vesicles (S), double vesicles (D), vesicles with double membrane (DM), multilayered vesicles (M), vesicle with broken membrane (B), granulated vesicle (GR). Scale bars are 100 nm.

**Fig 4:**
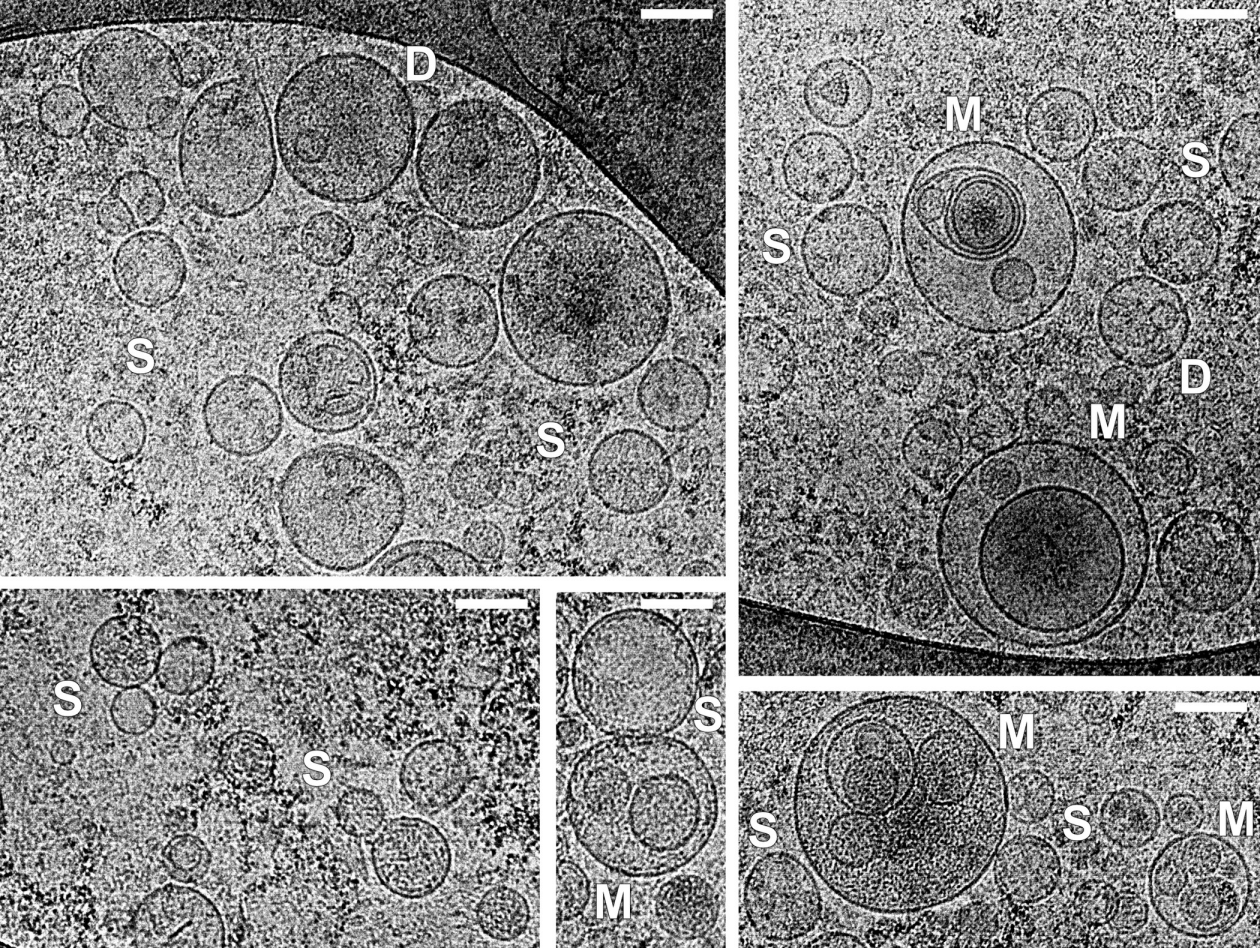
Cryo-EM images of EVs isolated from SAT and VAT culture mediums of individuals from the control group: Single (S), double (D), multilayered (M) vesicles. Scale bars are 100 nm.

The composition of EVs was heterogeneous including single layered vesicles, double membrane vesicles, double EVs (one inside the other), multilayered vesicles (two or more vesicles were contained inside a larger one), as well as exosomes (~100nm) and large vesicles (>100 nm) can be seen. Among EVs, single vesicles prevailed (up to 85% for SAT, up to 75% for VAT). Representative microphotographs of SAT and VAT EVs of obese patients with/without T2DM and individuals from the control group are presented in Figs 3 and 4, respectively. Additionally, microphotographs of EVs isolated from SAT and VAT culture mediums of patients with obesity and obese patients with concomitant T2DM are provided separately as supplementary material (S2–S5 Figs).

Some vesicles were more electron dense than others and among them vesicles were different from single to multilayered (more dark vesicles on Figs 3 and 4). One granulated EV with cargo was found in the sample of SAT EVs of patients with T2DM (Fig 3, tagged as GR: it had highly dense discrete inclusions inside).

**Fig 5:**
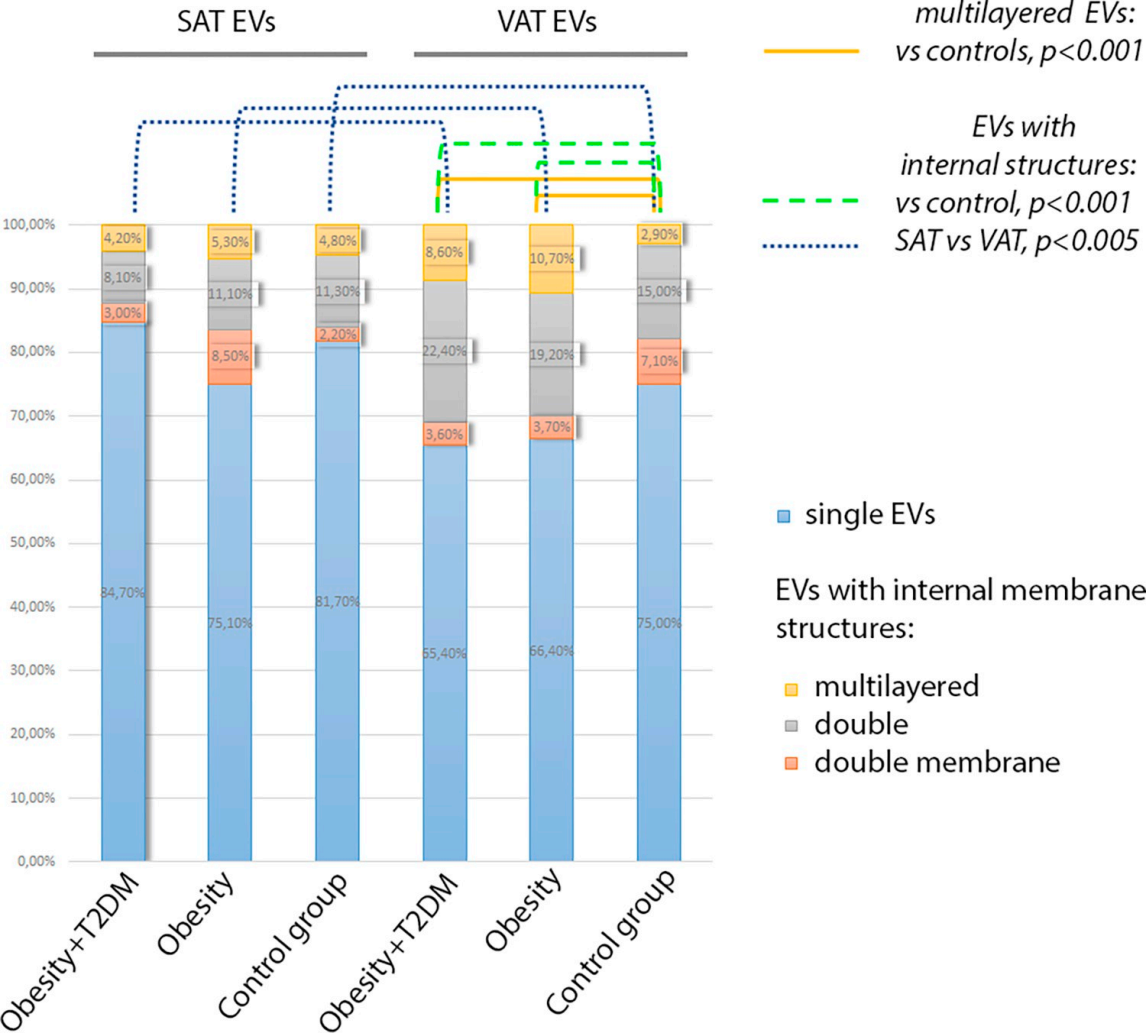
Proportional distribution diagram for main types of observed extracellular vesicles. Abbreviations in the figure: EVs–extracellular vesicles, SAT–subcutaneous adipose tissue, VAT–visceral adipose tissue, T2DM–type 2 diabetes mellitus.

The total proportion of EVs with internal membrane structures was higher in the samples of VAT EVs compared with the SAT EVs (p = 0.000 for the patient groups, p = 0.002 for the control group) (Fig 5). Obese patients with/without T2DM had larger proportion of multilayered EVs and EVs with internal structures for VAT when compared to the control group; there were no such differences between obese and control subjects in the case of SAT (Fig 5).

According to analyzed cryo-EM images mean size of single and double vesicles secreted by SAT were lower than VAT EVs for obese patients with and without T2DM (Table 3). Interestingly sizes of SAT and VAT EVs did not differ in the control group. Mean sizes of main types of observed vesicles are represented in Table 3.

**Table 3:** Sizes of main types of observed adipose tissue extracellular vesicles in the studied groups.

Group	Obesity with type 2 diabetes mellitus		*Obesity without type 2 diabetes mellitus*		Control group	
Adipose tissue type	SAT	VAT	SAT	VAT	SAT	VAT
Total number of analyzed EVs	334	532	434	494	1014	581
**Types of extracellular vesicles§:**						
***Single***						
Number, N(%)	283 (84,7%)	348 (65,4%)	326 (75,1%)	328 (66,4%)	828 (81,7%)	436 (75,0%)
Size, nm	52 (16–210)*	93 (25–275)	64 (19–261)*	89 (25–449)	82 (18–339)	76 (18–322)
***Double membrane***						
Number, N(%)	10 (3,0%)	19 (3,6%)	37 (8,5%)	18 (3,7%)	22 (2,2%)	41 (7,1%)
Size, nm	65 (49–116)	82 (43–277)	71 (38–130)¥	90 (58–142)	85 (51–154)	77 (32–184)
***Double (one inside the other)***						
Number, N(%)	27 (8,1%)	119 (22,4%)	48 (11,1%)	95 (19,2%)	115 (11,3%)	87 (15,0%)
Size, nm	97 (34–482)*	123 (35–298)	95 (40–211)**	148 (58–406)	121 (51–332)	109 (49–342)
***Multilayered***						
Number, N(%)	14 (4,2%)	46 (8,6%)	23 (5,3%)	53 (10,7%)	49 (4,8%)	17 (2,9%)
Size, nm	146 (92–381)	173 (122–446)	153 (64–431)¥	203 (119–347)	150 (72–311)	141 (76–225)
**Vesicles with defected or broken membrane:**						
Number	27	70	40	41	32	45
Size, nm	68 (29–177)	106 (40–374)	95 (34–308)	145 (63–378)	116 (45–274)	117 (35–327)

Notes

*p = 0,000 when compared with VAT EVs of corresponding group.

**p<0,01 when compared with VAT EVs of corresponding group.

¥ p<0,05 when compared with VAT EVs of corresponding group.

**§**Sizes of well-defined vesicles are given, including vesicles with slightly defected membranes, still allowing size measurement.

## Discussion

In this study EVs secreted by VAT and SAT of patients with obesity and T2DM for the first time were characterized by cryo-electron microscopy evaluating the morphology, size and phenotype.

Single, double, double with two membrane bilayers and multilayered vesicles were revealed in SAT and VAT EV preparations for all studied groups in the present study. All these types of EVs were demonstrated for other human biological fluids and cell culture conditioned medium using cryo-EM earlier as well as increased particle size of multilayered vesicles compared to single vesicles was shown [14–19]. We visualized double and multilayered vesicles containing electron dense material with varying degrees of intensity, while most vesicles were electron lucent as was described earlier [18,30]. In addition, elongated EVs and EVs with compromised membrane integrity were identified. That could be explained by possible deformation and breakage occurred during sample preparation. Indeed, some studies have demonstrated the effect of ultracentrifugation on the integrity and morphology of vesicles isolated by this method [30,31]. However, the variants of vesicle morphology observed in our study (double, multilayer, etc.) were also detected in a number of other studies during the isolation of EVs by alternative methods from various biological fluids [32–34].

Unexpectedly according to processed cryo-electron microphotographs single and double EVs from SAT tended to be smaller than from VAT in obesity and T2DM. The size of multilayered vesicles did not significantly differ between SAT and VAT as well as between groups. At the same time there were no differences in sizes of any EVs from SAT and VAT of control subjects. These features were not observed by NTA analysis for several reasons. First of all, NTA does not distinguish different types of EVs (monolayer/multilayer), additionally it does not reliably differentiate between vesicles and non-vesicular particulate material such as debris or protein aggregates, which leads to diameter of any particle that moves within the solution to be recorded [35]. NTA measures hydrodynamic rather than true particle diameter [19,36]. Determined size depends on refractive index of the particles and the smallest (under 50 nm) particles are worse detected by NTA, so they can be underestimated [19,37]. These technical points as well as lack of discrimination between populations could explain the difference in relative estimation of vesicles’ sizes in the NTA and cryo-EM data [35]. For example, the smallest observed multilayered vesicle was 64 nm which is comparable to the size of single vesicles.

Obesity affects the biogenesis of EVs: their production is increased during obesity and is correlated with the onset of obesity-related pathologies such as insulin resistance [38,39]. It could be speculated that reduced mean size of single and double SAT EVs may be a consequence of increased or accelerated secretion of smaller vesicles by SAT in obesity and associated metabolic complications. Recent studies showed that small vesicles (20–50 nm) of unclear vesicular nature and subcellular origin are present within EV preparations [25,37,40,41]. However, these smallest particles were shown to contain CD63, a representative MVB marker, on their membrane structure [40]. Another interesting point from our study is an increased proportion of multilayered vesicles from VAT compared to SAT that we observed for patients with obesity with and without T2DM. This may also indicate that the EV biogenesis pathway may be disrupted during obesity and visceral fat accumulation. Still the regulatory mechanisms controlling secretion of different classes of EVs are largely unknown [40]. MVBs are formed by invagination of vesicles into the endosome lumen to form intraluminal vesicles (ILVs), the process is mediated by the endosomal sorting complexes required for transport (ESCRT) machinery and by alternative way via tetraspanin webs and lipids rafts [42]. An additional mechanism for EV biogenesis involves the bioactive lipid ceramide which accumulates in the endosomal membrane and form lipid patches (similar to lipid rafts) that interact with proteins [42]. Adipocyte hypertrophy is associated with cellular stress and chronic low-grade inflammation which could regulate the secretion of EVs [39]. Additionally several important lipid molecules including ceramide are shown to be elevated in the adipose tissue of obese subjects which could influence the budding of MVBs from the adipocytes [43,44]. It has been proposed that ceramide promotes spontaneous membrane curvature by microdomain formation and coalescence into larger ceramide-rich domains, which onwards induce vesicle budding [39,45]. It was shown that EV secretion is markedly reduced by GW4869, a neutral inhibitor of sphingomyelinase, enzyme which generates the bioactive ceramide [46]. This is consistent with the data of demonstrated increased size of blood plasma EVs in patients with Gaucher disease which is caused by mutations in the gene encoding lysosomal enzyme glucocerebrosidase and characterized by accumulation of glucosylceramide [47]. Any adipocyte-derived EV is predicted to contain a lipid droplet and its associated proteins, including adipocyte triglyceride lipase and perilipin 1 [11]. It was shown that adipose tissue from lean mice released ~ 1% of its lipid content per day via EVs ex vivo, and this rate more than doubles with obesity [11]. So, it could be assumed that lipid accumulation in obesity influences size and morphology of EVs. Thus, lipids not only serve as structural components of exosomal membranes but also play a role in EV formation and release.

Recent studies proposed a link between the AMP-activated protein kinase (AMPK) pathway and EV secretion [45]. AMPK is a cellular sensor of energy homeostasis being a central player in glucose and lipid metabolism. Reduction of AMPK activity is associated with obesity and insulin resistance and subsequent enhanced EV release [45,48]. AMPK activation reduced adipocyte-mediated exosome release [48]. Inactivation of AMPK by its inhibitor compound C as well as silencing of AMPKα1 subunit encoding gene in 3T3L1 adipocytes both results in increased EV secretion and simultaneous enhanced TSG101 protein levels [48]. TSG101 is a core component of ESCRT pathway and plays a role in recruiting the whole ESCRT machinery, formation of ILVs and grouping them within MVBs [49,50].

Multilayered exosome-like vesicles were not exclusive for obese AT and were observed among SAT and VAT EVs of the control group; these structures are a natural occurence as they were found earlier in plasma and semen of healthy individuals [14,18,32]. The role of these vesicles is unclear and it can be only speculated that they could have special function such as transportation of internal vesicles. At the same time increased formation of such membrane structures may be the result of a pathological disturbance in the process of EV biogenesis. Still, it is currently unknown how multilayered vesicles are formed. Similar morphology of the vesicles was described for unprocessed EVs from ejaculate that suggests these structures are unlikely to be artifacts of ultracentrifugation [32]. Additionally, multilayered vesicles were observed among cerebrospinal fluid EVs isolated by size-exclusion chromatography [19]. It is known that MVBs are eventually degraded by lysosomes or fused with plasma membrane and secreted as EVs [42]. Increased proportion of multilayered EVs could be explained by accelerated release of MVBs that designed but failed to degrade in lysosomes. An increased number of double and multilayer vesicles among plasma EVs from patients with Gaucher disease was shown by us earlier and could be linked to lysosome dysfunction [47]. In present study larger proportion of vesicles with internal membrane structures was shown for VAT compared to SAT and this is more likely linked to the differences between fat depots. SAT functions as a benign storage depot of fatty acids as triglycerides, while VAT expansion is associated with abdominal obesity and it’s adverse metabolic and inflammatory profile [51]. Proteome analysis of adipose tissue EVs in obesity showed that VAT EVs are enriched in proteins related to energy pathway and metabolism, while SAT EVs contain more proteins linked to signal transduction and communication [52].

Durcin et al demonstrated differences in proteomic content of small and large (>100 nm, vesicles released from mature 3T3-L1 adipocytes [38]. Restricted number of proteins included in small EVs may be linked to the specific sorting of exosomes from MVBs, which is known to be controlled by Rab GTPases and other proteins of ESCRTI [38]. At the same time the fraction of large EVs which expected to contain multilayered EVs shows larger diversity of proteins [38]. This in turn may be related to the ability of large vesicles to encompass more material, especially cytosolic proteins and membranous lipid-raft proteins caveolin 1 and flotillin 2 [38]. Large EVs are specifically enriched in metabolic enzymes (mainly of mitochondrial origin) and thus may influence metabolic pathways in recipient cells [38]. It could be assumed that functions of large as well as multilayered EVs can be very diverse and additional studies are needed.

## Conclusion

In conclusion, this study for the first time described the characteristics and high degree of morphological variability of AT EVs. Larger proportion of vesicles with internal membrane structures including multilayered EVs compared with SAT is a feature of VAT. Decreased size of single and double SAT EVs compared to VAT EVs, large proportion of multilayered EVs and all EVs with internal membrane structures secreted by VAT distinguished obese patients with/without T2DM from the control group.

## Supporting information

S1 Fig DLS analysis of unprocessed adipose tissue EVs and EV sample after CD63-positive particles depletion. (TIF)

S2 Fig Cryo-EM images of EVs isolated from SAT culture medium of obese patients with T2DM. Various morphological types of extracellular vesicles have been identified: Single vesicles (S), double vesicles (D), vesicles with double membrane (DM), multilayered vesicles (M), vesicle with broken membrane (B), granulated vesicle (GR). Scale bars are 100 nm.
(TIFF)

S3 Fig Cryo-EM images of EVs isolated from SAT culture medium of obese patients without T2DM. Various morphological types of extracellular vesicles have been identified: Single vesicles (S), double vesicles (D), vesicles with double membrane (DM), multilayered vesicles (M). Scale bars are 100 nm.
(TIFF)

S4 Fig Cryo-EM images of EVs isolated from VAT culture medium of obese patients with T2DM. Various morphological types of extracellular vesicles have been identified: Single vesicles (S), double vesicles (D), vesicles with double membrane (DM), multilayered vesicles (M), vesicle with broken membrane (B). Scale bars are 100 nm.
(TIFF)

S5 Fig Cryo-EM images of EVs isolated from VAT culture medium of obese patients without T2DM. Various morphological types of extracellular vesicles have been identified: Single vesicles (S), double vesicles (D), vesicles with double membrane (DM), multilayered vesicles (M), vesicle with broken membrane (B). Scale bars are 100 nm.
(TIFF)

S1 File Western blot raw images. (PDF)
