# Supplementary figures and images for: Cryo-electron microscopy of adipose tissue extracellular vesicles in obesity and type 2 diabetes mellitus
Source: PLoS One. 2023 Feb 24;18(2):e0279652. doi: 10.1371/journal.pone.0279652 (PMC10045588; doi:10.1371/journal.pone.0279652)

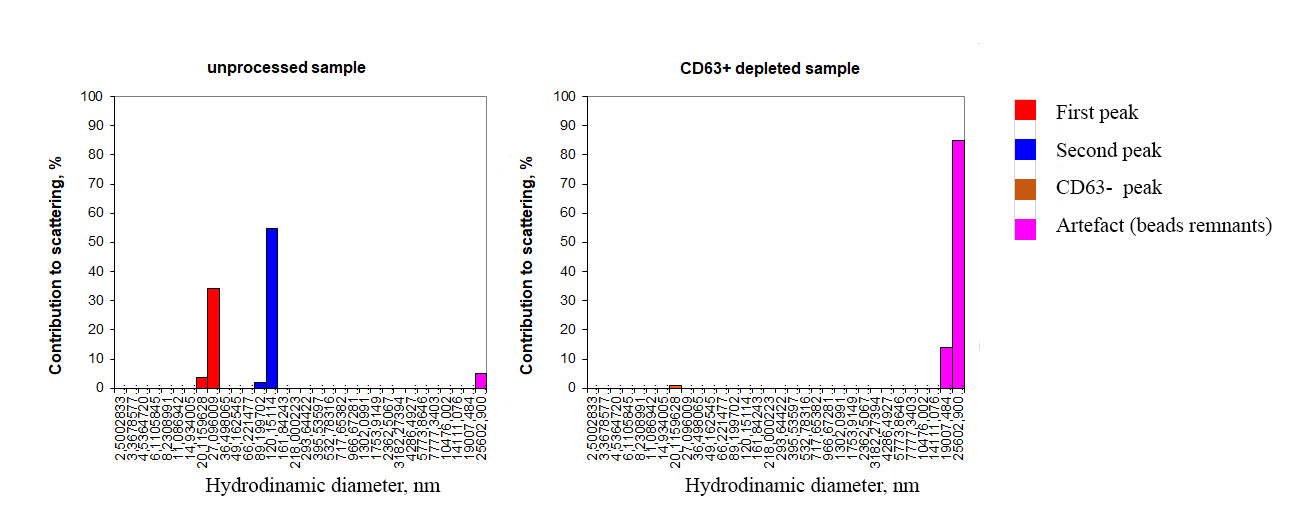

Supplement: S1 Fig — (TIF) [file pone.0279652.s001.tif]

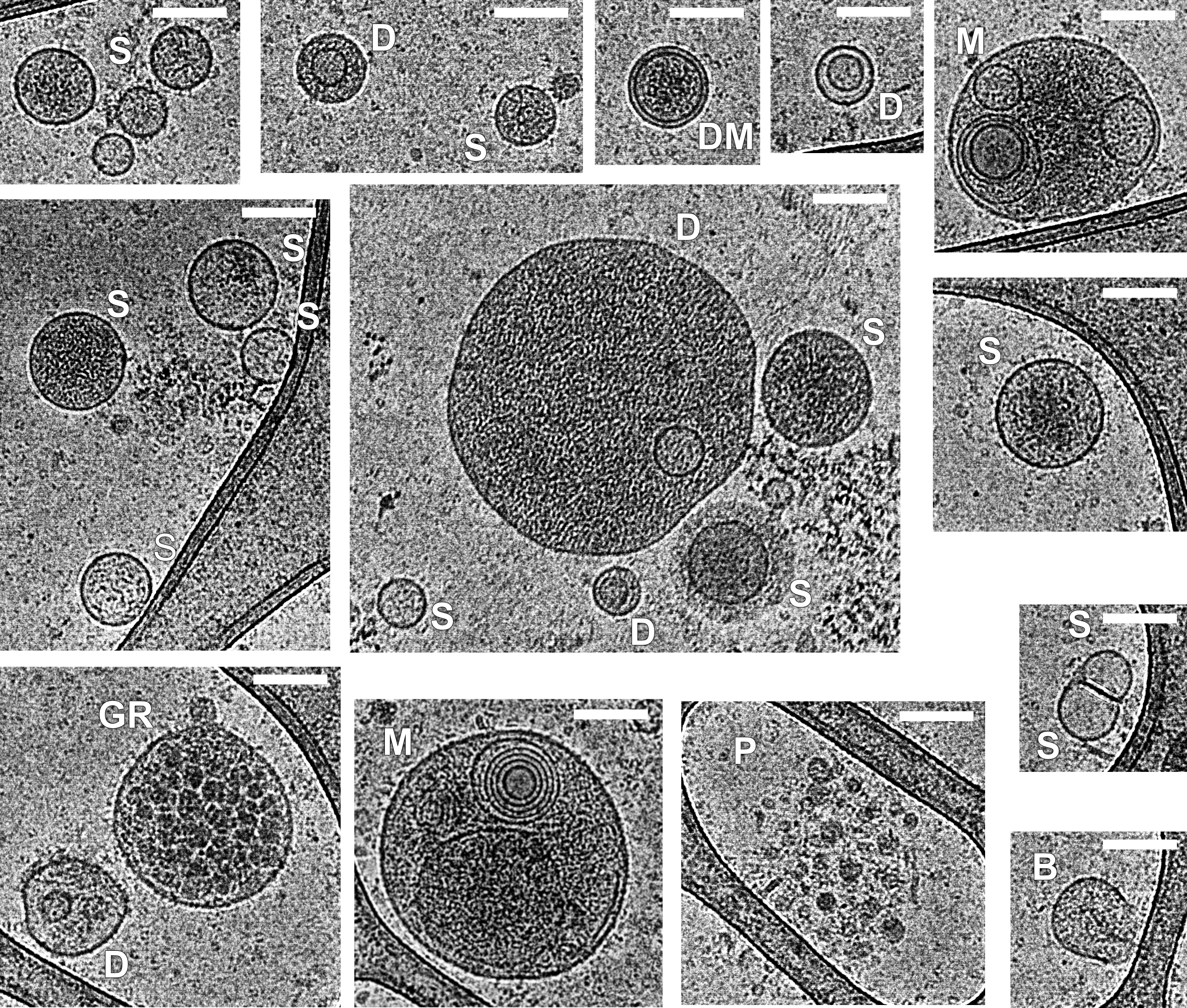

Supplement: S2 Fig — Various morphological types of extracellular vesicles have been identified: Single vesicles (S), double vesicles (D), vesicles with double membrane (DM), multilayered vesicles (M), vesicle with broken membrane (B), granulated vesicle (GR). Scale bars are 100 nm. (TIFF) [file pone.0279652.s002.tiff]

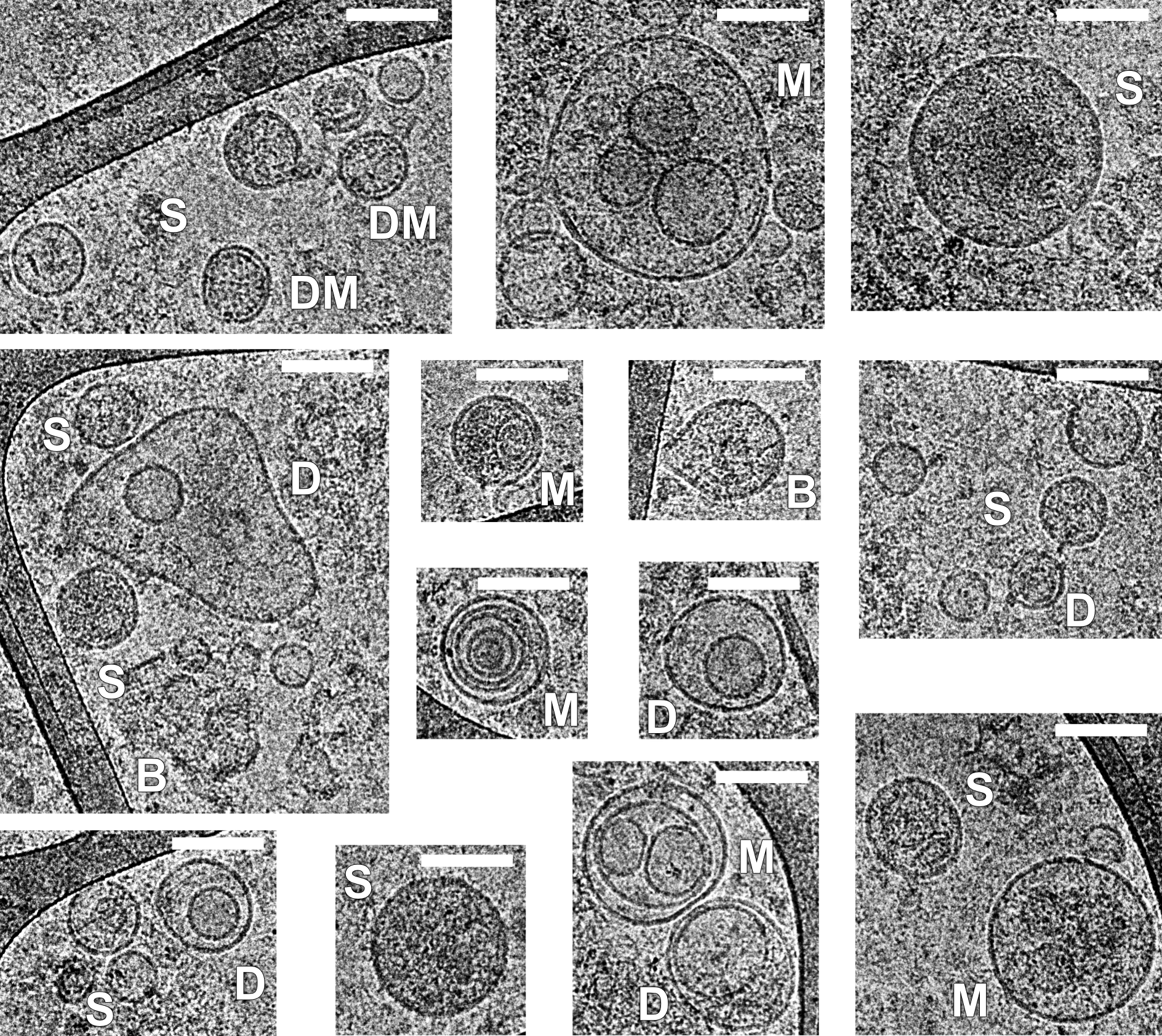

Supplement: S3 Fig — Various morphological types of extracellular vesicles have been identified: Single vesicles (S), double vesicles (D), vesicles with double membrane (DM), multilayered vesicles (M). Scale bars are 100 nm. (TIFF) [file pone.0279652.s003.tiff]

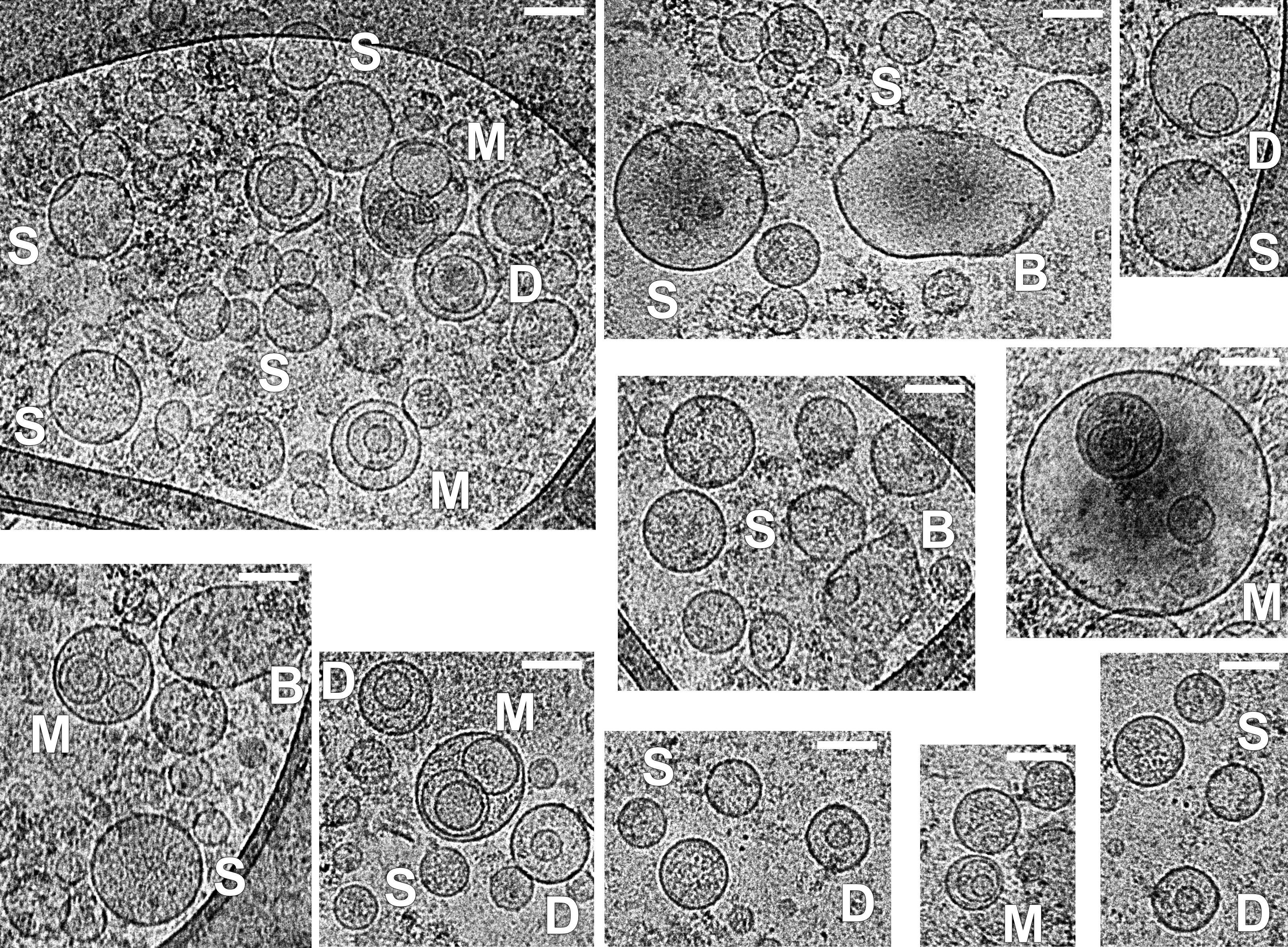

Supplement: S4 Fig — Various morphological types of extracellular vesicles have been identified: Single vesicles (S), double vesicles (D), vesicles with double membrane (DM), multilayered vesicles (M), vesicle with broken membrane (B). Scale bars are 100 nm. (TIFF) [file pone.0279652.s004.tiff]

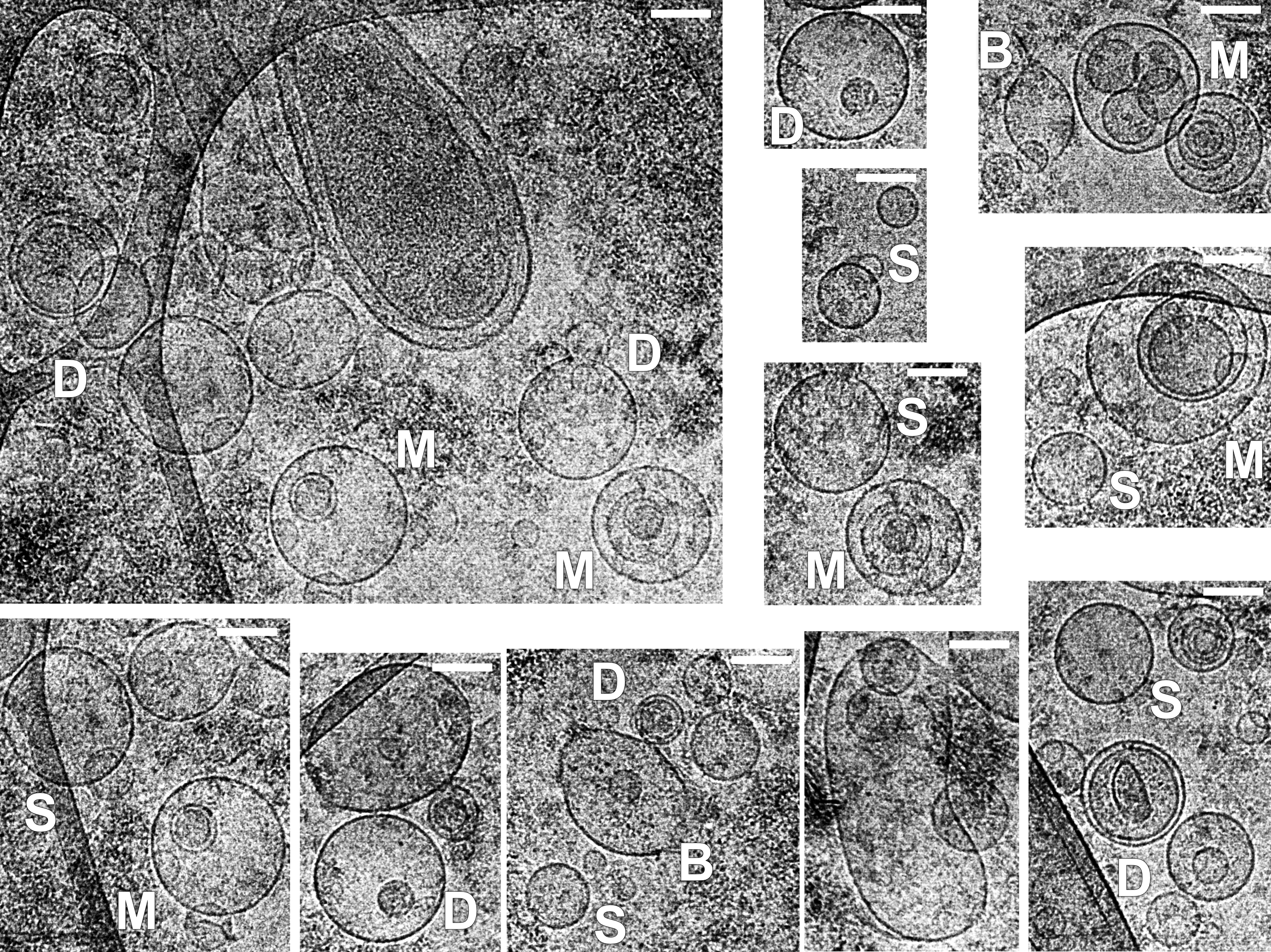

Supplement: S5 Fig — Various morphological types of extracellular vesicles have been identified: Single vesicles (S), double vesicles (D), vesicles with double membrane (DM), multilayered vesicles (M), vesicle with broken membrane (B). Scale bars are 100 nm. (TIFF) [file pone.0279652.s005.tiff]
