# Supplementary material for: Cryo-electron microscopy of adipose tissue extracellular vesicles in obesity and type 2 diabetes mellitus
Source: PLoS One. 2023 Feb 24;18(2):e0279652. doi: 10.1371/journal.pone.0279652 (PMC10045588; doi:10.1371/journal.pone.0279652)
Supplement: S1 File — (PDF) [file pone.0279652.s006.pdf]

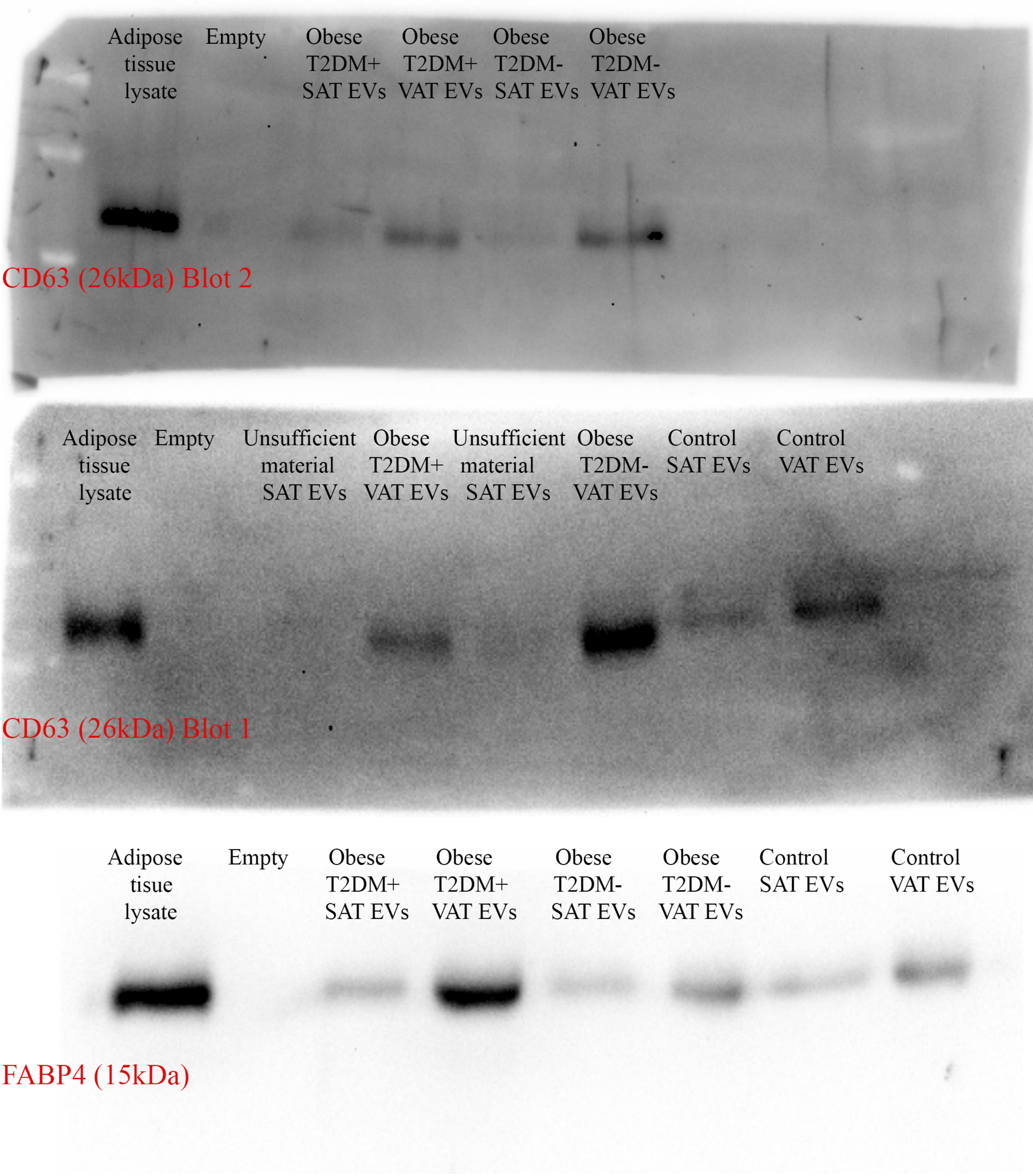
Adipose tissue lysate
Empty
Obese T2DM+ SAT EVs
Obese T2DM+ VAT EVs
Obese T2DM- SAT EVs
Obese T2DM- VAT EVs
CD63 (26kDa) Blot 2
Adipose tissue lysate
Empty
Unsufficient material SAT EVs
Obese T2DM+ VAT EVs
Unsufficient material SAT EVs
Obese T2DM- VAT EVs
Control SAT EVs
Control VAT EVs
CD63 (26kDa) Blot 1
Adipose tisue lysate
Empty
Obese T2DM+ SAT EVs
Obese T2DM+ VAT EVs
Obese T2DM- SAT EVs
Obese T2DM- VAT EVs
Control SAT EVs
Control VAT EVs
FABP4 (15kDa)
